# Investigating Measures of Social Context on 2 Population-Based Health Surveys, Hawaii, 2010–2012

**DOI:** 10.5888/pcd12.150319

**Published:** 2015-12-17

**Authors:** Ann M. Pobutsky, Kathleen Kromer Baker, Florentina Reyes-Salvail

**Affiliations:** Author Affiliations: Kathleen Kromer Baker, Florentina Reyes-Salvail, Hawaii State Department of Health, Honolulu, Hawaii.

## Abstract

Measures from the Social Context Module of the Centers for Disease Control and Prevention’s Behavioral Risk Factor Surveillance System were used on 2 population-based health surveys in Hawaii to explicate the role of the nonmedical and social determinants of health; these measures were also compared with conventional socioeconomic status (SES) variables. Results showed that the self-reported SES vulnerabilities of food and housing insecurity are both linked to demographic factors and physical and mental health status and significant when controlling for the conventional measures of SES. The social context module indicators should be increasingly used so results can inform appropriate interventions for vulnerable populations.

## Objective

Identifying differences in health status and social determinants of health (SDOH) such as socioeconomic status (SES) is fundamental to addressing health disparities (1). The Centers for Disease Control and Prevention’s (CDC’s) Behavioral Risk Factor Surveillance System (BRFSS) and the Hawaii Health Survey (HHS) measure the association of components of the SDOH through conventional demographic variables (ie, sex, race/ethnicity, education, or household income), but SDOH measures are becoming more established on cross-sectional health surveys (2–5). To measure SES vulnerabilities and health status in more detail, the Hawaii State Department of Health (DOH) used the CDC Social Context Module (6).

## Methods

The HHS and the BRFSS are annual landline and mobile telephone surveys used to assess risk for behaviors and health conditions associated with the leading causes of death. The Social Context Module includes measures of people’s perceptions of their SES vulnerabilities: worry about food security or payment for housing. The Social Context Module, which was added to the BRFSS in 2012, had 5,744 to 7,162 adult respondents (depending on the question). In 2010–2011 the module was added to the HHS, which had a sample size of 10,654 adult respondents.

Both data sets were weighted and used poststratification accounting for sampling design; the HHS also used a raking method to account for cellular telephones. The BRFSS data were not age-adjusted by race/ethnicity, and the HHS data were. The BRFSS and HHS analyses cover most but not all of the same variables. Poverty on the HHS was calculated by using US Department of Health and Human Services guidelines for 2011 (7). Tests of significant differences between prevalence values were performed with an α level of .05. The HHS used the mental component summary score from version 2 of the Short Form Health Survey (SF-12) (8) to compare associations with demographic characteristics and SES vulnerabilities; summary scores were standardized with a mean score of 50 and a standard deviation (SD) of 10 (lower scores represent poorer mental health). The Wald χ^2^ test was used for level and significance of associations. SAS version 9.4 (SAS Institute, Inc) and SUDAAN version 10.01 (RTI International) were used for all statistical analyses. Only the prevalence of food and housing insecurities on the HHS are shown by chronic conditions and risk factors (Table 1) along with the HHS regression analyses (Table 2). 

**Table 1:** Prevalence of Food and Housing Payment Insecurities in the Past 12 Months, by Demographic Characteristics (Age-Adjusted Rates), HHS, 2010 and 2011

Variable (Age-Adjusted)	Worried in the Past 12 Months About Having Enough Money to Buy Nutritious Meals		Worried in the Past 12 Months About Having Enough Money to Pay for Rent or Mortgage	
	Yes	No	Yes	No
	% (95% CL)			
Adults in Hawaii	29.1 (27.0–31.2)	70.9 (68.8–73.0)	46.0 (43.8–48.1)	54.0 (51.9–56.2)
**Fair/poor health**	43.8 (38.2–49.6)	56.2 (50.4–61.8)	57.2 (51.6–62.7)	42.8 (37.3–48.4)
**Mental health**				
Mental health score (median–50th quartile)	51.2 (50.2–53.3)	56.5 (55.7–57.1)	54.1 (52.3–54.3)	56.9 (55.9–57.1)
Pain limitations, moderate/extreme in last 4 weeks	47.8 (41.9–53.8)	52.2 (46.2–58.1)	62.1 (56.4–67.5)	37.9 (32.5–43.6)
Downhearted/blue all/most of time in last 4 weeks	51.4 (42.2–60.4)	48.6 (39.6–57.8)	63.3 (53.8–71.9)	36.7 (28.1–46.2)
**Physical health**				
Physical health score (median–50th quartile)	53.2 (52.1–53.5)	53.7 (53.5–54.4)	53.5 (52.9–53.6)	53.9 (53.5–54.5)
Physical limitations all/most last 4 weeks	44.0 (37.9–50.3)	56.0 (49.7–62.1)	55.3 (49.0–61.5)	44.7 (38.5–51.0)
**Current asthma**	35.7 (30.0–41.8)	64.3 (58.2–70.0)	56.2 (50.5–61.9)	43.8 (38.1–49.5)
**Diabetes**	49.4 (41.8–57.1)	50.6 (42.9–58.2)	65.7 (59.3–71.5)	34.3 (28.5–40.7)
**High blood cholesterol**	34.7 (28.7–41.2)	65.3 (58.8–71.3)	53.5 (47.7–59.2)	46.5 (40.8–52.3)
**High blood pressure**	31.8 (25.7–38.5)	68.2 (61.5–74.3)	55.0 (49.2–60.7)	45.0 (39.3–50.8)
**Heart disease**	41.9 (33.7–50.6)	58.1 (49.4–66.3)	53.2 (45.7–60.5)	46.8 (39.5–54.3)
**Lung disease**	39.9 (28.7–52.3)	60.1 (47.7–71.3)	54.6 (42.5–66.2)	45.4 (33.8–57.5)
**Stroke**	41.8 (33.2–51.1)	58.2 (48.9–66.8)	65.9 (51.5–77.9)	34.1 (22.1–48.5)
**Cancer**	40.2 (28.5–53.2)	59.8 (46.8–71.5)	50.6 (36.2–64.9)	49.4 (35.1–63.8)
**Obese**	35.9 (31.5–40.5)	64.1 (59.5–68.5)	54.5 (49.9–59.1)	45.5 (40.9–50.1)
**Underweight**	28.2 (20.8–37.1)	71.8 (62.9–79.2)	49.1 (40.4–57.8)	50.9 (42.2–59.6)

Abbreviations: HHS, Hawaii Health Survey; CL, confidence limit.

**Table 2:** Relationship Between SES and Demographic Variables and Mental Health Summary Score (SF-12), HHS, 2010 and 2011

Variable	Univariate		Multivariate^a^					
	Model 1		Model 2		Model 3		Model 4	
	Wald χ^2^	*P* Value	Wald χ^2^	*P* Value	Wald χ^2^	*P* Value	Wald χ^2^	*P* Value
Sex	8.5	.004	6.6	.01	4.3	.04	4.8	.03
Age	30.2	<.001	32.3	<.001	15.3	.002	15.2	.002
Race/ethnicity	17.9	.003	8.3	.14	8.0	.15	7.8	.17
Marital status	25.5	<.001	17.8	<.001	11.2	.001	15.3	<.001
High school graduate	22.7	<.001	29.1	<.001	23.1	<.001	23.4	<.001
Poverty	35.8	<.001	27.1	<.001	13.5	.001	17.7	<.001
Food worry	109.1	<.001	—	—	82.1	<.001	—	—
Rent worry	80.0	<.001	—	—	—	—	47.5	<.001

Abbreviations: —, not applicable; HHS, Hawaii Health Survey; SF-12, Short Form Health Survey 12.

a Adjusted for sex, age, race/ethnicity, marital status, high school graduate, and poverty.

## Results

All correlations differed significantly. According to BRFSS data (not shown), 1 in 5 people (21.7%) in Hawaii were worried in the past year about having enough money to buy nutritious food overall; this concern was higher among women (24.9%), adults younger than 65 years, residents of the neighboring islands of urban Oahu, adults who were previously or never married, people with lower education, and some ethnic groups (Native Hawaiians [31.5%], other Pacific Islanders [36.8%], and Filipinos [34%]). This same pattern was found among respondents who worried about paying their rent or mortgage in the past year, with one-third (32.7%) in Hawaii overall; the prevalence was higher among women, younger adults, people on the island of Hawaii, previously or never married adults, and people with lower education. By race/ethnicity, however, one-third or more of adults from all racial/ethnic groups except Japanese (the reference group) and Chinese reported worrying about paying their rent or mortgage. Adults who worried about paying for nutritious food were more likely to report being in fair or poor health (32.3%), as were those who worried about paying their rent or mortgage (46.3%). Adults who worried about paying for food or housing were more likely than those who were not worried to report having a chronic health condition: current asthma, chronic obstructive pulmonary disease, vision problems, or obesity. The HHS estimates (Table 1) on these measures are higher than those on the BRFSS (eg, on the HHS, 28.3% were worried about paying for food). The HHS results (Table 1) mirror the BRFSS results (not shown), except for the following notable differences: on the HHS, adults who worried about food or housing were more likely than those who were not worried to report having physical or pain limitations, diabetes, heart disease, stroke, obesity, lower mental health scores, or depression, compared to the BRFSS (data not shown). On the HHS, food and rent worries were highest among those near (38.5%) or below the poverty level (49%) (Table 1) (not measured on the BRFSS). Regression of perceived SES vulnerabilities as measured by the SF-12 on the HHS (Table 2) showed that, even while adjusting for demographic characteristics, the relationship between SES vulnerabilities and mental health/stress persists and is significant across all categories.

## Discussion

Using the BRFSS Social Context Module on 2 population-based surveys in Hawaii showed that SES vulnerabilities about paying for food or housing are associated with demographic characteristics, physical or mental health status, and some behavioral risk factors in Hawaii. Comparing results of the 2 surveys shows the same general patterns of perceived SES vulnerabilities on demographic characteristics, chronic conditions, and some risk factors. 

This study’s limitations consist of cross-sectional observations, which cannot provide evidence for causality, along with the potential recall or response bias, and recording or interviewer errors of telephone and cellular surveys and other self-reported data. There may be additional confounding variables that have not been controlled. Acknowledging that SES vulnerabilities affect health status can result in the development of policies and interventions that could be effective in reducing health disparities. These results also show the usefulness of adding social context and SES indicators to health surveys to enhance knowledge of the role of the social determinants on health. Increasingly, other states are also using these modules (9–12), along with others such as the industry and occupation module, to better assess the social determinants of health.
